# Severe hemophagocytic lymphohistiocytosis in a melanoma patient treated with ipilimumab + nivolumab

**DOI:** 10.1186/s40425-018-0384-0

**Published:** 2018-07-16

**Authors:** Andrew Hantel, Brooke Gabster, Jason X. Cheng, Harvey Golomb, Thomas F. Gajewski

**Affiliations:** 1 Department of Medicine, Section of Hematology/Oncology, The University of Chicago, 5841 S. Maryland Ave., MC2115, Chicago, IL 60637 USA; 2 Pritzker School of Medicine, The University of Chicago, Chicago, USA; 3 Department of Pathology, The University of Chicago, Chicago, USA

**Keywords:** Melanoma, HLH, Hemophagocytosis, Immune checkpoint, Checkpoint inhibitor

## Abstract

**Background:**

Treatment of metastatic melanoma patients with immune checkpoint inhibitors is an important standard of care. Side effects are due to immune activation, can affect virtually all organ systems, and are occasionally severe. Although hematologic toxicity has been reported, we present a case of hemophagocytic lymphohistiocytosis (HLH) due to immune checkpoint inhibitor therapy.

**Case presentation:**

A patient with metastatic melanoma was treated with one course of ipilimumab + nivolumab and presented 3 weeks later with severe anemia and hyperferritinemia. A bone marrow biopsy revealed necrotic tumor cells, infiltrating T cells, and hemophagocytosis. The patient was treated with high-dose steroids; 12 months later, the patient remains off all therapy and in complete remission of both HLH and metastatic melanoma.

**Conclusions:**

The hemophagocytic syndromes are attributable to dysregulated immune activation and share pathophysiologic mechanisms with immune activation from checkpoint inhibitors. Increasing use of regimens that include immune checkpoint inhibition require vigilant monitoring for immune-activating side effects as they can occasionally be life threatening, as in this case of HLH.

## Background

Hemophagocytic lymphohistiocytosis (HLH) is a severe and life-threatening condition of excess immune activation, inflammatory response, and multi-organ failure [1]. The hemophagocytic syndromes (HPS), of which HLH is a part, are a group of syndromes that can be broadly divided into genetic and acquired etiologies [2]. Familial HLH (FLH), also referred to as primary or inherited, occurs as a result of a gene mutation in either one of the FLH loci or one of several loci responsible for immunodeficiency syndromes. The secondary HPS are comprised of acquired HLH and the pathophysiologically identical macrophage activation syndrome (MAS) [3]. HLH can stem from a variety of predisposing conditions of immune dysregulation such as malignancy, infection, or acquired immunodeficiency. The term MAS is used only when secondary HPS is a complication of rheumatologic disease, most commonly with systemic juvenile idiopathic arthritis, systemic lupus erythematosis, or adult onset Still’s Disease [4]. HLH has been described, using various nomenclature, since a report 1939 by Scott and Smith [5] whereas MAS was first described in the literature in 1985 by Hadchouel [6].

The modern immunologic basis for HPS began to be uncovered in 1996 after the identification of cytotoxic deficiencies and common inflammatory patterns in patients with HLH [7]. This was followed shortly thereafter by the first description of perforin gene mutations in FLH by Stepp et al. [8]. In FLH, most known mutations result in protein deficiencies within the cytolytic secretory pathway. In this pathway, perforin and granzyme-containing granules are secreted into the synapses between cytolytic cells, namely cytotoxic T cells or natural killer cells, and their targets. Due to low cytolytic function, immune activation persists and hyperinflammation paradoxically results [9]. This pathophysiologic construct is clearer in FLH than in the acquired HLH, where abnormal T cell activation and inflammatory cytokine production, as well as paradoxical down-regulation of B cell function, Toll-like receptor expression and signaling, and apoptosis induction have also been noted to varying degrees [10, 11].

As a syndromic diagnosis, the classification of HLH was established by the Histiocyte Society in 1994 [12] and was most recently updated in their HLH-2004 guideline [3]. Unless molecular testing can establish a genetic basis for the syndrome, a constellation of eight criteria is used. To meet the diagnostic criteria, five of the following eight criteria must be met: fever (> 38 °C); splenomegaly; cytopenias affecting two or more cell lines (hemoglobin < 9 g/dL, platelets < 100 × 10^3^/mL, neutrophils < 1 × 10^3^/mL), hypertriglyceridemia (fasting, > 265 mg/dL) and/or hypofibrinogenemia (< 150 mg/dL), hemophagocytosis in bone marrow, spleen, lymph nodes, or liver; low or absent NK cell activity; ferritin > 500 ng/mL; elevated soluble CD25.

Immune checkpoint inhibition is the mainstay of modern treatment for metastatic melanoma [13]. Current checkpoint inhibitor therapy in melanoma is based on two immune targets: cytotoxic T-lymphocyte antigen 4 (CTLA-4) and the programmed cell death 1 (PD-1). CTLA-4 is a transmembrane protein expressed by activated CD4^+^ and CD8^+^ T-cells and negatively regulates their activation by antigen presenting cells [14]. PD-1 is a transmembrane protein expressed on activated T cells, B cells, and NK cells that also inhibits their function upon engagement of the ligand, PD-ligand 1 (PDL-1), which is found across many tissue types including tumor cells [14]. PD-1/PDL-1 interactions have been associated with T cell dysfunction in the tumor microenvironment, and also can play a role in the conversion of conventional T cells to regulatory T-cells [15]. Antibodies against these targets have been shown to improve T cell activation and exert anti-tumor immunity in multiple preclinical models, including augmented cytolytic T cell activity [16]. Notably, these are the characteristics of immune responses that appear to be dysregulated in HLH.

Antibodies targeting CLTA-4 and PD-1, ipilimumab and nivolumab or pembrolizumab, have been independently shown to improve overall survival in unresectable melanoma [17–19]. More recently, their use in combination was found to improve response rates, performance free survival, and overall survival in the first line setting [20]. The Checkmate-067 Trial studied the combination of nivolumab and ipilimumab versus either nivolumab or ipilimumab as single agents. In a recently updated analysis, the trial showed objective response rates of 58.9, 44.6, and 19%, median progression-free survival of 11.7 months, 6.9 months, and 2.9 months, and 3 year overall survival rates of 58, 52, and 34% for nivolumab plus ipilimumab, nivolumab, and ipilimumab, respectively. Notable within the original report of the Checkmate-067 Trial was the increased incidence of grade 3 or 4 side effects in the combination treatment group at 58.5% vs 20.8% with nivolumab and 27.7% with ipilimumab [21]. Additionally, close to 40% of patients on combination therapy stopped treatment due to adverse events.

There appears to be significant overlap between the dysregulated immune activation underlying HLH and the immune activating toxicities seen with immune checkpoint inhibitors. Below, we present our case of HLH in the setting of metastatic melanoma treated with combination checkpoint inhibition.

## Case presentation

A 35-year-old woman was evaluated in clinic with progressive fatigue, pre-syncope, upper respiratory symptoms, pallor, and low-grade fevers over the preceding week. She had been diagnosed with locally advanced malignant melanoma of her left upper extremity 10 months prior, in March 2016. The patient had undergone a complete surgical resection with sentinel lymph node biopsy, which showed melanoma with a Breslow depth of 2.2 mm. One of three sentinel nodes was positive for metastasis and the tumor was negative for BRAF or KIT mutations. This resection was followed by a complete level three axillary lymph node dissection, which was negative for further metastasis. She then received 4 cycles of adjuvant ipilimumab, which was complicated by panhypopituitarism. Two months after completing adjuvant treatment she reported a dry cough and fatigue. Computed tomography showed widespread metastatic disease in the bilateral lungs and axial skeleton, including a sternal mass with soft tissue extension. Three weeks before presentation she was given her first doses of combination immunotherapy with ipilimumab and nivolumab.

On examination, she was pale and slightly jaundiced. Cardiac exam revealed a regular, tachycardic rhythm and her lungs were clear to auscultation. She had a benign abdominal exam but exhibited palpable splenomegaly below the left costal margin. There were no ecchymoses or petechiae. Her heart rate was 121 BPM and her blood pressure was 82/45 mmHg. Her hematologic work up revealed a hemoglobin of 2.9 mg/dL and a platelet count of 79 × 10^3/uL, both decreased from values of 8.0 mg/dL and 119 × 10^3/uL, respectively, 2 weeks prior. Her bilirubin was elevated to 2.9 mg/dL, of which 1.7 mg/dL was unconjugated. Her prothrombin and partial prothrombin times were normal and the patient denied any recent history of bleeding. She was given intravenous fluid boluses and admitted to the hospital for further work-up.

**Fig. 1:**
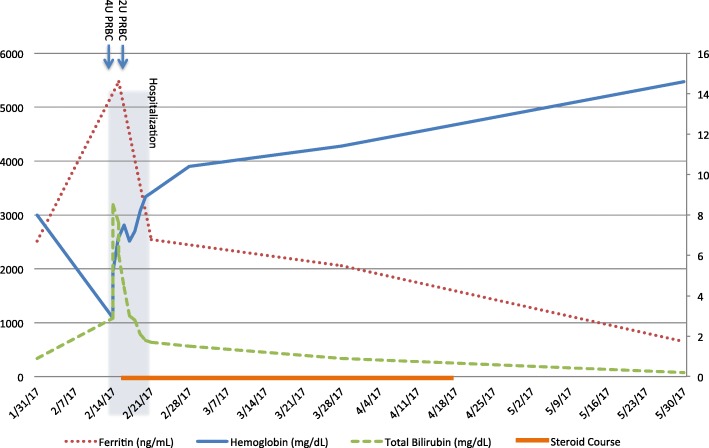
Laboratory value trends over time. Time of hospitalization is shaded. Units of blood are marked correspondingly as “PRBC.” Period of steroid administration is noted along the X-axis. Ferritin, hemoglobin, and bilirubin levels are noted

In the hospital, her hemoglobin level increased to 7.0 mg/dL after being transfused with 4 units of packed red blood cells. It decreased again below 7.0 mg/dL on hospital days two and three, prompting two more units of blood. Further laboratory testing revealed a lactate dehydrogenase level of 1029 U/L, a C-reactive protein of 202 mg/L, and a haptoglobin of < 20 mg/dL. Her direct Coombs test was negative and glucose-6-phosphate dehydrogenase level was normal. Bilirubin increased to 8.5 mg/dL, of which 5.4 mg/dL was conjugated. Her ferritin level was elevated to 5474 ng/mL and fasting triglyceride level was 336 mg/dL. The reticulocyte production index was 0.6. An ADAMTS13 function was normal and an inhibitor was not found. Her cortisol level was 42.7 μg/dL, thyrotropin was reduced at 0.07 mcU/mL, and free thyroxine was 1.65 ng/dL. An infectious work up, including blood and urine cultures, parvovirus, EBV, and CMV serology was negative. An MRI of her liver showed a stable hemangioma and stable metastatic deposits were noted in the incidentally visible lung. Trends in the patient’s laboratory values can be seen in Fig. 1.

**Fig. 2:**
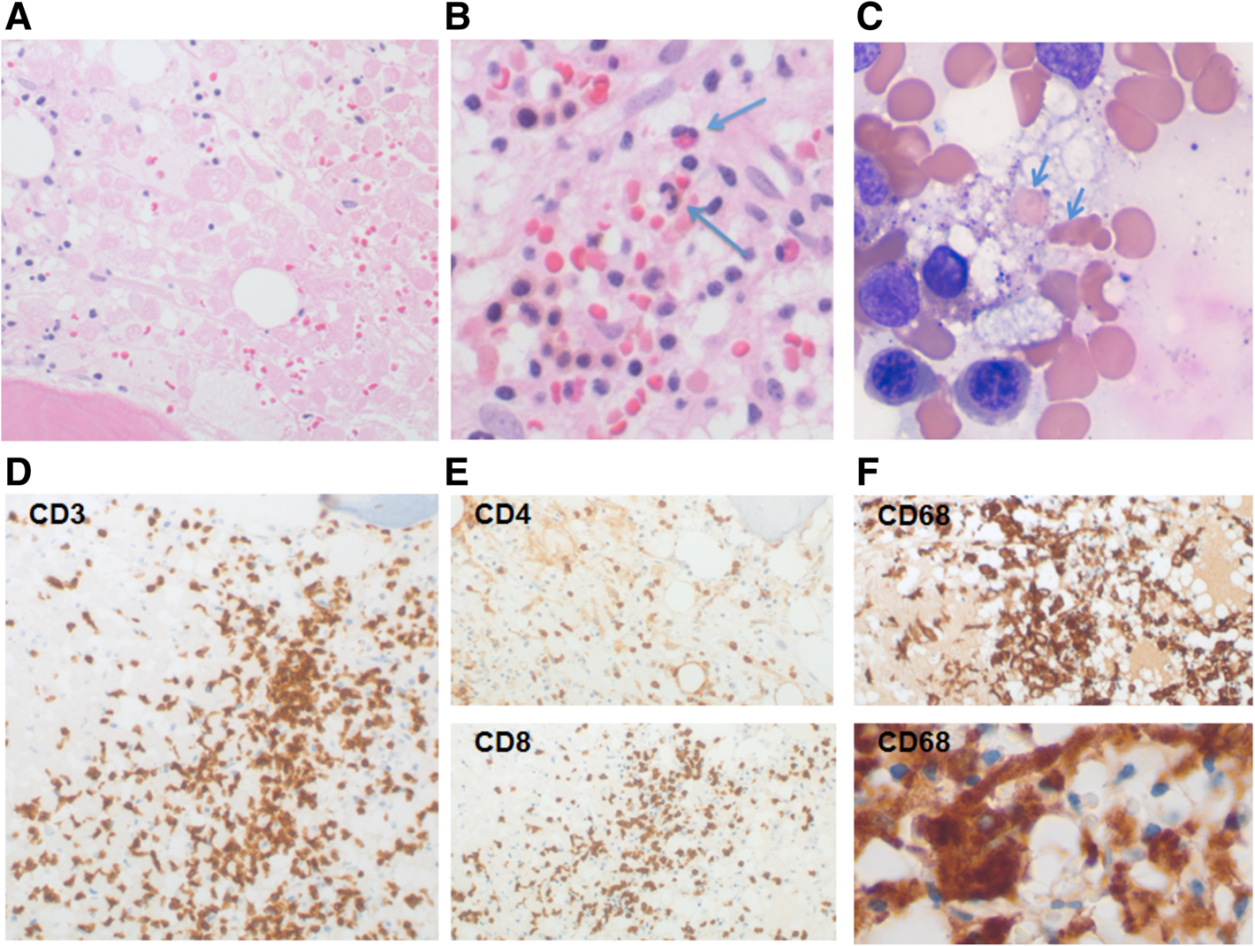
Bone marrow biopsy obtained at diagnosis. **a**. Necrotic melanoma cells; H&E, magnification of 500×. **b**. Phagocytosing histiocytes containing red blood cells; H&E, magnification of 500×, blue arrows. **c**. Phagocytosing histiocytes containing red blood cells, higher magnification. D-F. Immunohistochemistry for CD3 (D), CD8 and CD4 (E), and CD68 (F); magnification of 200×

A hematopathology review of the peripheral blood showed a marked reduction in red blood cells with the remainder displaying spherocytosis, ansiopoikilcytosis, and rare teardrop forms. The granulocytes and lymphocytes appeared mature and normal. A bone marrow biopsy of her posterior iliac crest was performed. The core biopsy sections (Fig. 2) revealed a hypercellular bone marrow (approximately 85% of total area) with large sections of tumor cell necrosis. Occasional positive S100 and Melan A immunostains were noted within the dead tumor debris. CD3^+^ T cells were abundant, the majority of which were CD8^+^ (Fig. 2c). Moderate serous atrophy and MF-2 fibrosis was noted on reticulin staining. Unexpectedly, it also revealed increased histiocytes throughout the marrow with active hemophagocytosis (Fig. 2b, blue arrows). Soluble CD25 levels were significantly elevated at 2840 U/mL.

Given these laboratory results, in combination with the patient’s clinical signs and symptoms, HLH was considered. The patient met HLH-2004 criteria with her splenomegaly, cytopenias, hypertriglyceridemia, hemophagocytosis, elevated ferritin, and elevated soluble CD25 levels establishing the diagnosis. The patient was started on 1.5 mg/kg of methylprednisone every 8 hours on hospital day four and her hemoglobin began to improve; further transfusions were not required. She was monitored in the hospital for 4 days and the steroid dose was decreased to 1 mg/kg of oral prednisone. On discharge, her hemoglobin had increased to 9.1 mg/dL without further transfusion. The use of additional treatment, per HLH-2004 guidelines, was considered but was decided against given her marked improvement and with consideration of the etiology of her presentation.

**Fig. 3:**
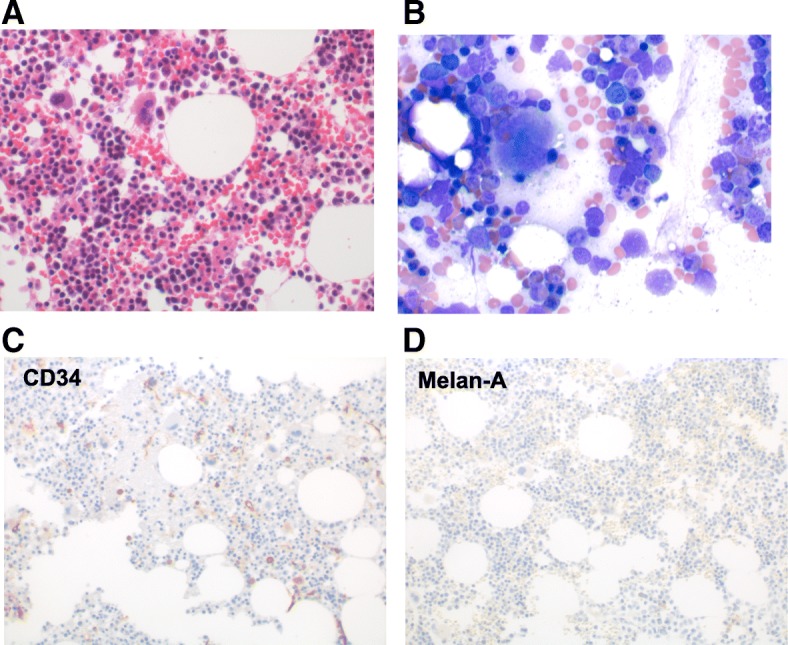
Bone marrow biopsy obtained after disease resolution. **a** Regenerative BM core, H&E, magnification of 500×. **b** BM aspirate smears showing erythroid hyperplasia with no neoplastic cells. Wright-Giemsa, magnification of 500×. Immunohistochemistry for CD34 (**c**) and Melan-A (**d**). There was no increase in CD34^+^ blasts, and no detectable Melan-A-positive cells, i.e. no melanoma; magnification 200×

Over the course of the next 2 months, her bilirubin decreased to 0.2 mg/dL, ferritin to 651, CRP to < 3 mg/L, and lactate dehydrogenase to 253 U/L (Fig. 1). She remained on high-dose steroids for 1 month before being tapered slowly down to her prior replacement dose regimen for panhypopituitarism. On a follow up clinic visits in 2017, the hemoglobin was maintained above 14 mg/dL and a computed tomographic scans revealed a resolution of both her previous metastatic lung nodules and sternal mass. A repeat bone marrow biopsy was performed due to a residual abnormal bone marrow intensity noted by CT imaging. This demonstrated focal bone marrow remodeling with trilineage hematopoiesis, no hemophagocytic activity, and no morphologic or immunohistochemical evidence of involvement by metastatic melanoma (Fig. 3). The patient remains off treatment and in complete remission 12 months later.

## Discussion and conclusions

As anti-CTLA-4 and PD-1 antibodies lead to immune activation, inflammatory effects can occur in any organ system [22]. The majority of patients on single-agent immunotherapy do not experience any side effects and the most common side effects are rash and mild fatigue, likely related to increased circulating cytokines [23]. When specific immune-related toxicity occurs, frequently affected systems include the endocrine organs, gastrointestinal tract, integument, and lungs. Thus, in additional to history and physical exam, thyroid function studies, complete blood counts, and metabolic panels including liver function are generally checked at each treatment and at intervals of 6 to 12 weeks for the first 6 months after finishing treatment, when the risk of toxicity is highest [23, 24]. In patients with non-specific symptoms such as fatigue, evaluation of adrenocorticotropic hormone, cortisol, follicle-stimulating hormone, luteinizing hormone, prolactin, testosterone in men, and estradiol in women is typically pursued. Treatment with corticosteroids regimens, such as intravenous methylprednisolone 1–2 mg/kg per day for 3–5 days, followed by prednisone, 1–2 mg/kg per day gradually tapered over 4 weeks can reverse the vast majority of toxic manifestations of these drugs; prolonged tapers are recommended as shorter tapers can lead to a recurrence of symptoms or adrenal crisis [24]. Generally, systemic corticosteroids are used only for grade 3 to 4 or prolonged grade 2 immune-related side effects.

Serious immune-mediated hematologic side effects have also been noted with checkpoint blockade antibodies. Several cases of autoimmune hemolytic anemia have been seen after treatment with nivolumab [25–28]. All of these cases were direct antibody test (DAT) positive for C3 or IgG, did not involve other cytopenias, and most responded to corticosteroids. Pure red cell aplasia has also been seen after treatment with pembrolizumab; while one case of this responded to a prolonged course of corticosteroids, another required further treatments to respond [29, 30]. This second patient developed germinal center-like reactions within the bone marrow that eventually resolved with IVIG followed by the anti-CD20 antibody rituximab [29]. Anti-platelet antibody-positive idiopathic thrombocytopenic purpura has also been seen as a result of anti-PD-1 antibody therapy [31, 32]. In one case, increased PD-1 expression prior to nivolumab treatment was found on the patient’s B cells. Treatment of these patients also required intravenous immune globulin and in one case the thrombopoetin analogue romiplostin was administered. While not reported with anti-PD-1 antibody therapy, metastatic melanoma patients treated with IL-2 have been observed to develope thrombotic thrombocytopenic purpura, each case requiring cessation of therapy and treatment with plasmapheresis [33]. Many patients in this group of hematologic complications had prior auto-immune like toxicities with therapy, including hepatitis, hypothyroidism, and pneumonitis [24–32]. We are not aware of a previous case of hemophagocytic lymphohistiocytosis reported as a complication of immune therapy for cancer.

Our patient met the criteria for HLH with splenomegaly, cytopenias, hypertriglyceridemia, hemophagocytosis, elevated ferritin, and elevated soluble CD25 levels. Except for her recent treatment with ipilimumab and nivolumab, there was no other obvious or common cause of HLH including fulminant progression of disease or overwhelming infection. Additionally, other similarly presenting diagnoses, including disseminated intravascular coagulation, autoimmune hemolytic anemia, thrombotic thrombocytopenic purpura, or liver failure, were effectively ruled out. Given that the immunologic processes governing both the pathophysiology of HLH and the beneficial effects of checkpoint blockade are similar, future cases of immunotherapy complicated by DAT-negative anemia and low reticulocyte indices should prompt consideration of the HPS syndromes.

Outcomes for the HPS syndromes vary depending on the underlying cause [1]. Given the germline predisposition of FLH, long-term remission in these patients requires allogeneic stem cell transplantation. Treatment of secondary HLH generally follows the HLH-2004 protocol with etoposide and corticosteroids as well as therapy specific to the underlying disease (antibiotics, additional chemotherapy, etc.) [3]. In our patient, both the significant elevation in bilirubin as well as the excellent response to corticosteroids in immunologic events considered secondary to checkpoint blockade dissuaded us from the use of up-front etoposide. Fortunately, she responded both clinically and by laboratory values to corticosteroids alone and her HLH has not recrudesced to date. If corticosteroids alone were not sufficient, our treatment team would have considered IL-1R antagonist or anti-IL-6 antibodies, which have been used successfully in childhood cases of MAS given the clear immunologic nature of the etiology in the current case.

This is, to our knowledge, the first reported experience of immunotherapy with checkpoint blockade causing hemophagocytic lymphocytosis. The trigger for the syndrome in this case may have been related to rapid tumor cell death and immune activation specifically within the bone marrow compartment, as evidenced by the tumor cell debris along side of CD8^+^ T cell infiltration and hemophagocytosis seen by bone marrow biopsy. Recently, Gordon et al. published a report demonstrating that tumor-associated macrophages increase over time in mouse and human models of cancer and that PD-1 expression inversely relates to their phagocytic potency against tumor cells [34]. With the addition of blockade against PD-1/PDL-1, they showed an increase in macrophage phagocytosis of tumor cells. While this study did not demonstrate increased hemophagocytosis or specific effects on histiocyte activity, it is an intriguing biological rationale for the increased phagocytic activity seen in this case. Future care of cancer patients treated with these agents should consider HLH if similar constellations of symptoms and laboratory values arise.
